# *Omanicotyle heterospina* n. gen. *et* n. comb. (Monogenea: Microcotylidae) from the gills of *Argyrops spinifer* (Forsskål) (Teleostei: Sparidae) from the Sea of Oman

**DOI:** 10.1186/1756-3305-6-170

**Published:** 2013-06-07

**Authors:** Gil Ha Yoon, Sarah Al-Jufaili, Mark A Freeman, James E Bron, Giuseppe Paladini, Andrew P Shinn

**Affiliations:** 1 Department of Marine Science & Fisheries, College of Agricultural & Marine Sciences, Sultan Qaboos University, P.O. Box 34, Al-Khod 123, Oman; 2 Ministry of Agriculture and Fisheries, P.O. Box 427, Muscat 100, Oman; 3 Institute of Ocean and Earth Sciences, University of Malaya, Kuala Lumpur 50603, Malaysia; 4 Institute of Aquaculture, School of Natural Sciences, University of Stirling, Stirling FK9 4LA, UK

## Abstract

**Background:**

The Sultanate of Oman’s aquaculture industry is expanding with an on-going assessment of potential new fish species for culture. The king soldier bream, *Argyrops spinifer* (Forsskål) (Sparidae), is one such species that is under consideration. During a routine health assessment of specimens caught in the Sea of Oman throughout the period November 2009 to March 2011, a number of gill polyopisthocotylean monogeneans were recovered.

**Methods:**

A subsequent study of the monogeneans using a range of morphology-based approaches indicated that these were *Bivagina heterospina* Mamaev *et* Parukhin, 1974. In the absence of pre-existing molecular data, an expanded description of this species is provided, including a differential diagnosis with other species and genera belonging to the subfamily Microcotylinae Monticelli, 1892 with the subsequent movement of this species to a new genus to accommodate it.

**Results:**

The polyopisthocotyleans collected from the gills of *A. spinifer* appear to be unique within the family Microcotylidae Taschenberg, 1879 in that, morphologically, they possess a pair of large, muscular vaginae each armed with a full crown of 16–18 robust spines and a unique dorsal region of folded tegument, which permits their discrimination from species of *Bivagina* Yamaguti, 1963. Sequencing of the SSU rDNA (complete 1968 bp) and LSU rDNA (partial 949 bp) places the specimens collected during this study within the subfamily Microcotylinae, but the LSU rDNA sequence differs from *Bivagina* and also from other microcotylid genera. Morphological features of *B. heterospina sensu* Mamaev *et* Parukhin, 1974 and the specimens collected from the current study are consistent with one another and represent a single species. The vaginal armature of these worms is unique and differs from all other genera within the Microcotylinae, including *Bivagina*, and its movement to *Omanicotyle* n. gen. to accommodate this species is proposed.

**Conclusions:**

A new genus, *Omanicotyle* n. gen., is erected to accommodate *Omanicotyle* [*Bivagina*] *heterospina* n. comb. which represents the first monogenean to be described from Omani marine waters. Given the pathogenic potential of microcotylids on captive held fish stocks, a full assessment of *Omanicotyle heterospina* n. gen. *et* n. comb. is now required before large-scale production commences.

## Background

In addition to the ~150,000 tonnes of commercially important species of wild marine fish landed through Omani ports [1], the Omani aquaculture industry, which currently exceeds 500 tonnes p.a. and primarily concerns the production of *Dicentrarchus labrax* (L.) and *Oreochromis niloticus niloticus* (L.) [1], is exploring the potential of a number of native species suitable for aquaculture. Of the 990+ fish species known from Omani waters [2], approximately 50 species are under consideration for use in aquaculture, which will include an assessment of various species of seabream, grouper and snapper. Although global aquaculture production is expected to rise to meet the shortfall in wild catches, there is a parallel requirement to identify potential threats to the health and welfare of wild fisheries and aquaculture stocks so that contingencies to mitigate against their establishment or to minimise their potential impacts can be taken. During a recent assessment of these new aquaculture species by the Ministry of Fisheries Wealth, a sample of king soldier bream, *Argyrops spinifer* (Forsskål), was found to be infected with a microcotylid polyopisthocotylean monogenean, whose morphology is consistent with *Bivagina heterospina* Mamaev *et* Parukhin, 1974, which was previously described from the same host from Mokura Bay, Kuria Muria Islands in the Arabian Sea [3]. The morphological features of *B. heterospina*, however, appear unique and differ from other species of the genus *Bivagina* Yamaguti, 1963 and an expanded description of the microcotylid is provided in the current study. A new genus to accommodate this species is proposed.

## Methods

### Collection of host and parasite material

Thirty specimens of *Argyrops spinifer*, with a standard length of 26–52 cm, were collected by line angling in the Oman Sea off the coastal city of Muscat and landed at Muttrah (23° 37′ 08.65″ N; 58° 35′ 33.76 E) throughout the period November 2009 to June 2010. After landing, the fish were placed on ice and then directly transferred to the Ministry of Fisheries Wealth where the gills were excised. A total of 18 adult monogeneans (mean intensity of infection 0.6 ± 1.0 parasites fish^-1^; range 0–3) parasitising the gills, were removed using mounted surgical needles. Fifteen of the specimens were transferred directly into 90% ethanol for subsequent evaluation by staining, scanning electron and confocal laser scanning microscopy. The remaining three specimens were fixed in 95% ethanol for molecular studies.

### Morphological methods

Whole mounts of specimens (n = 7) were stained with Mayer’s paracarmine to highlight features of the internal anatomy. Specimens were measured using an eyepiece graticule and all measurements are given in micrometres as the range followed by the mean in parentheses, unless otherwise stated. The terminology of structures follows that of Williams [4]; lengths refer to measurements taken along the longitudinal axis of the worm.

Additional ethanol fixed specimens (n = 5) were prepared for scanning electron microscopy by rehydrating down through a graded ethanol series to water and then by transferring them into 0.2 M sodium cacodylate buffer pH 8 for 24 h, post-fixed in 1% osmium tetroxide for 2 hours at room temperature and then dehydrated through a graded ethanol series. Specimens were then transferred to 50:50 100% ethanol: hexamethyldisilazane followed by 2 changes of 100% hexamethyldisilazane (45 min each), air dried overnight, mounted on 12.5 mm aluminium stubs (Agar Scientific Ltd., Stansted, UK) and then sputter‒coated with gold using an Edwards Sputter Coater S150B. Specimens were viewed under a Jeol JSM 6460 LV SEM, at an accelerating voltage of 10 kV.

A further three alcohol fixed specimens were processed for confocal laser scanning microscopy, by rinsing them in distilled water for 24 h and then transferring them to either 1) 40 mM chromotrope 2R (C2R) (Alfa Aesar, Heysham, UK) + 3 mM phosphotungstic acid (Sigma-Aldrich®, Poole, UK) + 0.5% acetic acid (Sigma-Aldrich®) for 4 h at room temperature [5] to stain the attachment clamps and copulatory spines; or 2) 5 μl (0.2U μl^-1^ methanol) Alexa Fluor® 594 phalloidin (Invitrogen Molecular Probes, Eugene, Oregon, USA) in 100 μl distilled water in the dark, at room temperature for 5 h to stain the muscular components of the worm. After staining, the specimens were rinsed and mounted in distilled water and then examined on a Leica TCS SP2 AOBS laser scanning confocal microscope.

Illustrations were prepared from images captured using a Zeiss AxioCam MRc digital camera mounted on top of an Olympus BX51 compound microscope using a ×0.75 interfacing lens and ×10 to ×100 oil immersion objectives and MRGrab 1.0.0.4 (Carl Zeiss Vision GmbH, 2001) software.

### Molecular methods

Three individual monogeneans, previously fixed in 95% ethanol, were digested overnight at 56°C in DNA buffer containing 100 μg ml^-1^ proteinase K. Total DNA was extracted using a GeneMATRIX kit (EURx Poland) following the tissue protocol and used for PCR reactions. The small subunit ribosomal DNA (SSU rDNA) was amplified using the primers 18e, 390f, 870f/r and 18gM [6-9]. The D1-D2 domains of the large subunit ribosomal DNA (LSU rDNA) were amplified using the primers C1 and D2 [10]. PCR bands of the correct size were visualised and recovered from the PCR products using a GeneMATRIX PCR products extraction kit (EURx Poland). PCR reactions were performed in triplicate (three separate worms) according to the original descriptions and sequencing reactions were performed using BigDyeTM Terminator Cycle Sequencing chemistry utilising the same oligonucleotide primers used for the original PCRs. DNA sequencing was performed in both directions for all PCR products and contiguous sequences obtained manually using CLUSTAL_X [11] and BioEdit [12]. CLUSTAL_X was used for the initial sequence alignments and regions of ambiguous sequence alignments were manually edited using the BioEdit sequence alignment editor [12]. Alignment files of related microcotylids, consisting of 947 characters of LSU rDNA sequence data, were used in the phylogenetic analyses. Phylogenetic analyses were performed using the maximum likelihood methodology in PhyML [13] with the general time-reversible (GTR) substitution model selected and 1000 bootstrap repeats, and Bayesian inference (BI) analyses using MrBayes v. 3.0 [14]. Models of nucleotide substitution were evaluated for the data using MrModeltest v. 2.2 [15]. The most parameter-rich evolutionary model based on the Akaike Information Criterion (AIC) was the GTR + I + G (GTR + proportion Invariant + Gamma) model of evolution. Posterior probability distributions were generated using the Markov Chain Monte Carlo (MCMC) method with four chains being run simultaneously for 1,000,000 generations. Burn in was set at 2500 and trees were sampled every 100 generations making a total of 7500 trees used to compile the majority rule consensus trees.

Taxa used in the phylogenetic analysis with respective GenBank accession numbers in parentheses: *Atrispinum acarne* Maillard *et* Noisy, 1979 (AF311702); *Bivagina pagrosomi* (Murray, 1931) Dillon *et* Hargis, 1965 (AJ243678); *Cynoscionicola* “*branquialis*” (AF382050); *Diplostamenides sciaenae* (Goto, 1894) Lebedev, Parukhin *et* Roitman, 1970 (FJ432589); *Microcotyle arripis* Sandars, 1945 (GU263830); *Microcotyle erythrinii* van Beneden *et* Hesse, 1863 (AM157221); *Microcotyle sebastis* Goto, 1894 (AF382051); *Pagellicotyle mormyri* (Lorenz, 1878) Mamaev, 1984 (AF311713); *Polylabris sillaginae* (Woolcock, 1936) Dillon, Hargis *et* Harrises, 1983 (GU289509); *Sparicotyle chrysophryii* (van Beneden *et* Hesse, 1863) Mamaev, 1984 (AF311719).

### Type material examined

The following museum type specimens were examined: 1 paratype (CNHE 203) and 8 voucher specimens (CNHE 2822, 2824, 2827) of *Pseudobivagina aniversaria* (Bravo-Hollis, 1979) Mamaev, 1986 (syn. *Neobivagina aniversaria* Bravo-Hollis, 1979) from the Cortez sea chub *Khyposus elegans* (Peters) from the Colección Nacional de Helmintos, Instituto de Biologia, Universidad Nacional Autónoma de México, Mexico City, Mexico; 3 voucher specimens of *Bivagina pagrosomi* (NHM 1980.6.2.6-7) from the gills of the silver seabream *Pagrus* (syn. *Chrysophrys*) *auratus* (Forster); 4 voucher specimens of *Microcotyle* [labelled as *Bivagina*] *centrodonti* Brown, 1929 (NHM 1989.4.28.33-52) from the gills of the black seabream *Spondyliosoma cantharus* (L.); and 1 voucher specimen of *Bivagina* sp. (NHM 1985.11.8.27) from the gills of the red stumpnose bream *Chrysoblephus gibbiceps* (Valenciennes) from the national parasite collection maintained by the Parasites and Vectors Section, The Natural History Museum, London, UK. In addition, photographs of *Bivagina tai* (Yamaguti, 1938) from the gills of red sea bream *Pagrus major* (Temminck *et* Schlegel) were kindly provided for assessment by Professor Kazuo Ogawa from the Meguro Parasitological Museum, Tokyo, Japan. Although valuable type material of *B. heterospina* were not available for loan, photographs of paratype (acc. no. 12296) from the gills of *A. spinifer* caught in Mokura Bay, Kuria Muria Islands in the Arabian Sea on the 26^th^ August 1969 were generously provided by Dr Pavel Gerasev from the Zoological Institute of The Russian Academy of Sciences (ZIRAS), St Petersburg, Russia.

## Results

Class Monogenea Carus, 1863

Family Microcotylidae Taschenberg, 1879

Subfamily Microcotylinae Monticelli, 1892

***Omanicotyle*** n. gen.

### Diagnosis

Body lanceolate. Haptor symmetrical, with numerous clamps arranged in two equal rows. Clamps of microcotylid type of approximate equal size. No terminal anchors present. Paired muscular, unarmed, septate buccal organs. Oesophagus simple without diverticula. Intestinal crura not symmetrical, largely co-extensive with vitellaria extending into the haptoral peduncle. Testes numerous, post-ovarian. Genital atrium muscular, unarmed. Two large, paired, muscular, dorsal, vaginae, each armed with a crown of robust equal sized spines. Germarium U-shaped. Germinal part of germarium approximately ovoid. Large, fusiform, operculated eggs with two polar filaments, an extensive apical filament and a short posterior filament. Conspicuous Y-shaped vitelline duct. Vitellaria extending into the haptor.

*Type species: Omanicotyle heterospina* (Mamaev *et* Parukhin, 1974) n. comb.

*Etymology:* The generic name refers to the type locality, Oman.

***Omanicotyle heterospina*** (Mamaev *et* Parukhin, 1974) n. comb.

Syn. *Bivagina heterospina* Mamaev *et* Parukhin, 1974.

*Type host:* King soldier bream, *Argyrops spinifer* (Forsskål) (Sparidae); wild (current study; [3]).

*Other hosts:* Soldierbream, *Argyrops filamentosus* (Valenciennes) [3].

*Site on the host:* Gills.

*Type locality:* Kuria Muria Islands, Arabian Sea (17° 28′ 06.14″ N; 55° 35′ 55.18″ E) [3].

*Other localities:* Sea of Oman landed at the port of Muttrah (23° 37′ 08.65″ N; 58° 35′ 33.76 E) (current study).

*Type material:* Holotype (acc. no. 226/IO-1628) deposited in the Helminthology Laboratory of General Biology and Soil Science of the USSR FESC. Paratypes (acc. no. 12296) are stored in the Laboratory of Parasitology of the Zoological Institute of the USSR.

*Voucher material:* Seven Mayer’s paracarmine stained whole mounts prepared for the current study. Three voucher specimens (acc. no. NHMUK 2013.5.13.1-3) are deposited in the parasite collection of the Parasites and Vectors Section, The Natural History Museum (NHM), London. A further two specimens (USNPC acc. no. 106952.00) are deposited in the United States National Parasite Collection (USNPC), Beltsville, MD, USA; and two additional voucher specimens (acc. no. AHC 35684) are deposited in the Australian Helminthological Collection (AHC) of The South Australian Museum (SAMA), North Terrace, Adelaide.

*Molecular sequence data:* A complete SSU rDNA sequence of 1968 bp and a partial LSU rDNA sequence of 949 bp have been deposited in GenBank under the accession numbers JN602094 (SSU) and JN602095 (LSU), respectively.

*General:* To comply with the regulations set out in article 8.5 of the amended 2012 version of the International Code of Zoological Nomenclature (ICZN) [16], details of this species have been submitted to ZooBank with the Life Science Identifier (LSID) zoobank.org:pub: CDEC0135-BA59-4754-B213-5C748A02C818. In addition, a species profile including taxonomic traits, host details and additional metadata are provided on http://www.monodb.org[17].

**Figure 1:**
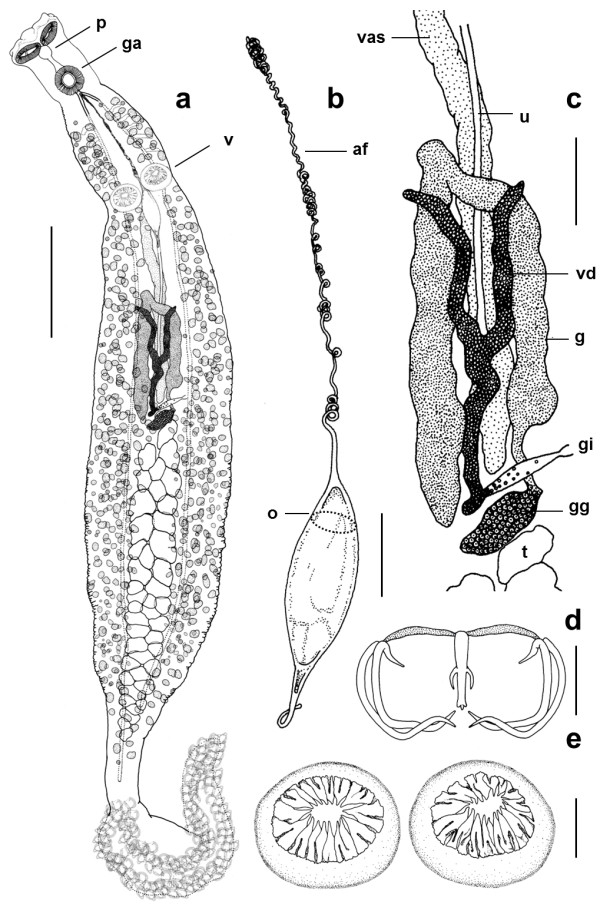
***Omanicotyle heterospina *n. gen. *et *n. comb. recovered from the gills of *Argyrops spinife r *(Forsskål). a**, whole mount: ga, genital atrium; p, pharynx; v, vaginae. The vitellaria and pigment granules extend into the haptor but are not shown in full as they would obscure other body features. **b**, egg: af, anterior filament; o, approximate position of the operculum. **c**, reproductive system: g, germarium; gg, germinal part of germarium; gi, genito-intestinal canal; t, testes; u, uterus; vas, *vas deferens*; vd, vitelline duct. **d**, clamp. **e**, paired, armed vaginae. Scale bars: a = 500 μm; b, c = 100 μm; d = 25 μm; e = 50 μm.

**Figure 2:**
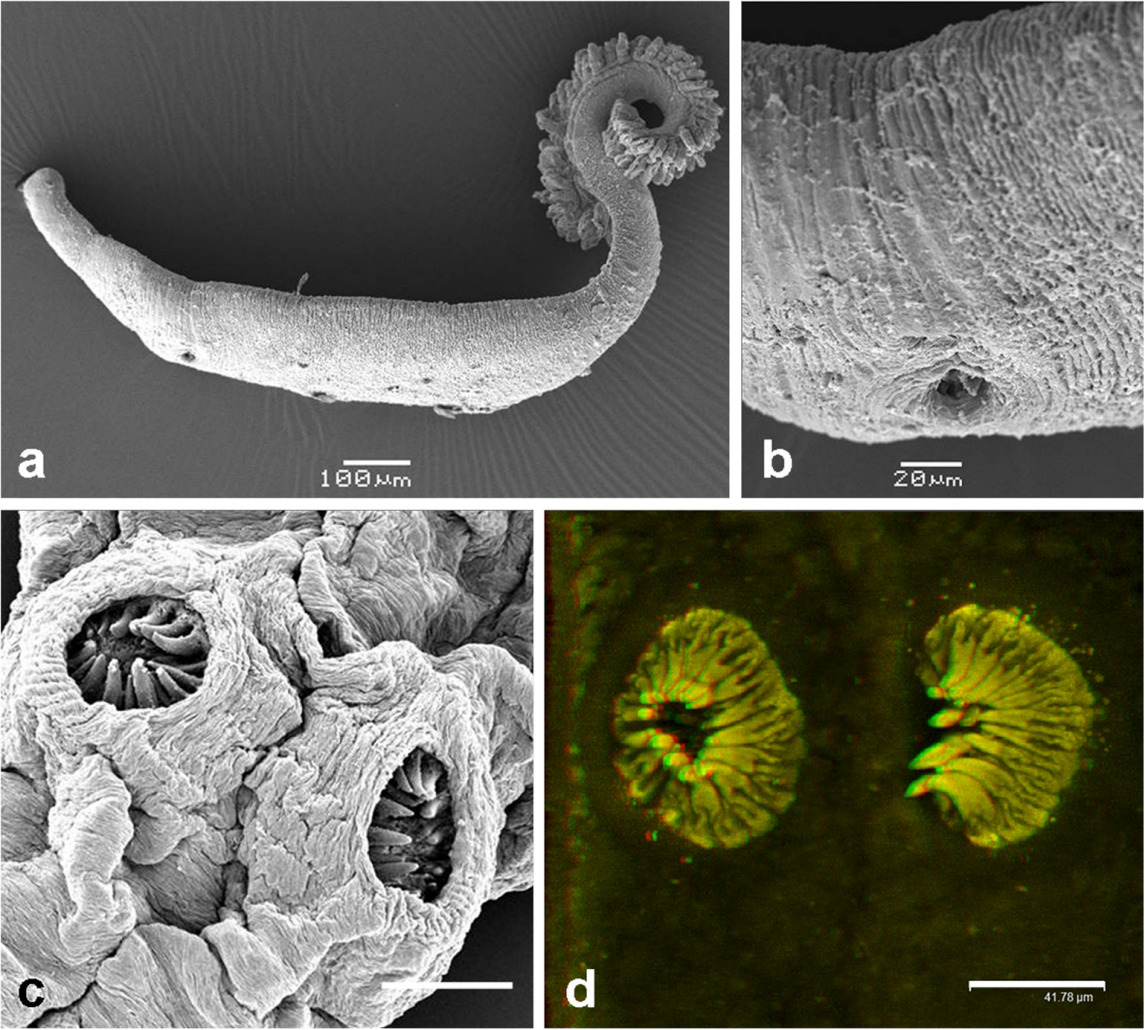
***Omanicotyle heterospina *n. gen. *et *n. comb. from the gills of *Argyrops spinifer *(Forsskål). a**, Scanning electron micrograph (SEM) of an entire specimen. **b**, close up of the opening of one of the paired, dorsally positioned vaginae. **c**, desiccated specimen prepared for SEM to reveal the crown of 16–18 spines within each vagina. **d**, laser scanning confocal microscope image of the vaginal spines stained with a chromotrope 2R based stain (see [4]). Scale bars: a = 100 μm; b = 20 μm; c = 50 μm; d = 41.8 μm.

**Figure 3:**
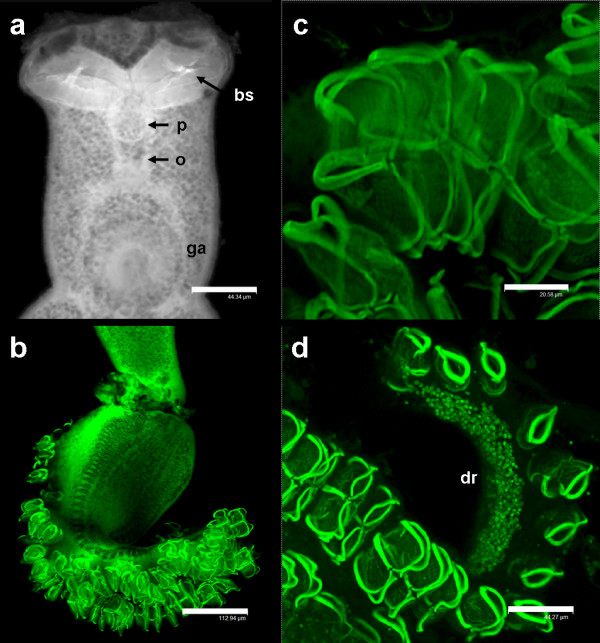
**Laser scanning confocal micrographs of the anterior region and haptor of *Omanicotyle heterospina *n. gen. *et *n. comb. a**, Muscular elements of the anterior region stained with Alexa Fluor® 594 phalloidin. The septum of each buccal sucker (bs) is clearly evident. The lower edge of each sucker appears to be serrated or crenulated by contraction of the fine muscles in this region but are not spine bearing. The buccal cavity leads to a muscular circular pharynx (p), a short oesophagus (o) and an unarmed genital atrium (ga). **b**, oblique view of the symmetrical haptor which bears up to 50 pairs of clamps arranged in two equal rows. The vitellaria and pigment granules are visible as two parallel, dark, patterned bands running the length of the haptor. **c**, dual staining of the attachment clamps with phalloidin and the chromotrope 2R-based stain to reveal the main sclerotised component and the associated musculature. **d**, anterior, forward projecting section of the haptor stained with 40 mM chromotrope 2R (C2R) + 3 mM phosphotungstic acid + 0.5% acetic acid for 4 h at room temperature. The photograph shows that clamps develop in a posterior to anterior direction and the lateral margins of this region of the haptor bear numerous C2R positive droplets (dr). Scale bars: a = 44.34 μm; b = 112.94 μm; c = 20.58 μm; d = 44.27 μm.

**Figure 4:**
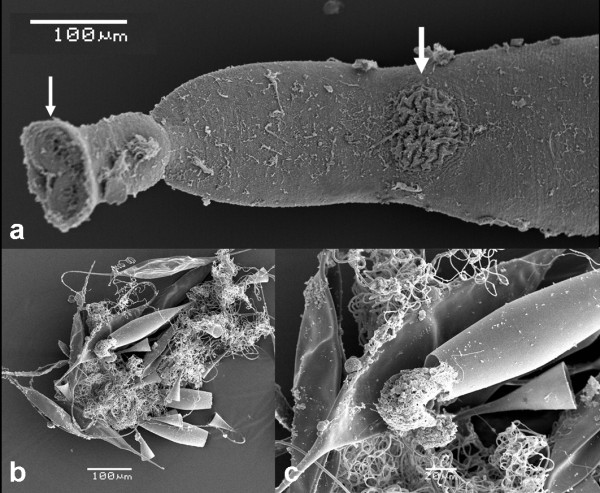
**Scanning electron micrographs of *Omanicotyle heterospina *n. gen. *et *n. comb. a**, The anterior end of the worm, showing two septate buccal suckers (thin arrow), and its delineation from the main body. A circular area of irregular folded tegument on the dorsal surface is also evident (thick arrow), the precise function of which is unknown. **b**, the spindle shaped, operculated eggs, each bearing a short posterior filament and a long, tangled, apical filament which may exceed 10× the length of the egg proper. **c**, a ciliated oncomiracidium emerging from an egg. Scale bars: a, b = 100 μm; c = 20 μm.

**Description** (based on 7 mature whole mounts): Body elongate with deep lateral constriction posterior to the genital atrium, a second lateral constriction in the vicinity of the vaginae and a narrow peduncle leading into the haptor (Figure 1). Total body length, including haptor, 4826 (2750–7375); 593 (275–775) in maximum width at the level of the germarium. Haptor symmetrical, delineated from the body, triangular, containing 45–50 pairs of clamps arranged in two equal rows (Figures 1a, 2a, and 3b). Lateral margins of the anterior part of the haptor well developed in its anterior part, the margins of which contain numerous C2R positive droplets (Figure 3d). Clamps, of approximate similar shape but size slightly dissimilar; clamps develop in a posterior to anterior direction (Figure 3b-d). Anterior clamps 71 (68–75) wide, 36 (28–43) long; median clamps 76 (70–80) wide, 30 (28–38) long; posterior clamps 58 (48–65) wide, 34 (28–40) long. Haptoral hooks absent. Circular area of folded tegument on the dorsal surface, posterior to the vaginae approximately 70 long × 90 wide (Figure 4a). This irregular folding of the tegument was present on all 7 specimens mounted for light microscopy; function unknown. Structure evident in specimens prepared for SEM (see Figure 4a). Anterior region containing the buccal organs, pharynx and genital atrium, delineated from the main body by a sharp narrowing of the worm (Figures 1a, 2a, and 3a). Paired muscular buccal organs lacking discernible spines, 137 (113–158) long, 79 (45–100) wide, with septum 36 (25–60) long (Figure 3a); buccal cavity leading to a muscular circular pharynx 44 (40–48) long, 44 (35–53) wide. Oesophagus 124 (100–175) long. Intestinal caecae not equal in length, one terminates post-testicular, the other extends into the peduncle terminating just before the haptor. Short diverticula present in the anterior section posterior to the genital atrium, not evident throughout its entire length as obscured by vitellaria. Vitellaria irregular, not well defined, yellowish-brown in colouration, co-extensive with intestinal caecae, extending from the genital atrium to mid-way along the haptoral peduncle, each granule 40 (20–60) long, 21 (18–25) wide. Pigment granules distributed throughout the body, a light scattering anterior to the vaginae, below this they concentrate into two dark, lateral bands that run the length of the body extending into and running the length of the haptor. Genital atrium unarmed, 77 (68–93) wide, 74 (63–88) long at its base; projects away from the body as a small cone. Two large, muscular vaginae 110 (90–130) wide, 120 (100–128) long, each armed with a crown of 16–18 robust spines, 24–28 long, which curve slightly towards the centre of the structure (Figures 1a,e and 2c,d). The muscles forming the wall of each vagina, 28 (25–30) thick. Worm narrows at mid-level of vaginae. Testes 42 (35–47) in number, situated in post-ovarian intercaecal field, not extending into the haptoral penduncle. U-shaped germarium median, pre-testicular, 754 (675–850) total length; 406 (337–550) length of the distal portion; 348 (275–475) length of the proximal part (excluding the germinal part of the germarium); germinal part of the germarium 96 (75–113) length × 55 (43–63) width (observed in 6 specimens). Vitelline ducts Y-shaped, branches 191 (185–200) long, the posterior piece 242 (230–255) long, which leads into the genito-intestinal canal opening above the germinal part of the germarium. Fusiform, operculated eggs 230 long, 80 wide (present in 4 specimens). Very long, tangled, apical filament ~1060 long, which tapers towards its extremity, the terminus of which is not thickened; posterior filament 110 long (Figures 1b, c and 4b,c).

### Remarks

The original specimens of *Omanicotyle* [*Bivagina*] *heterospina* (Mamaev *et* Parukhin, 1974) n. comb. were collected from Kuria Muria Islands in the Arabian Sea, off the coast of Oman in July-August 1967 and in August-September 1969. This represents a separate water body, as recognised by the International Hydrographic Organisation, to the material collected from the Sea of Oman for the current study. There are, however, some small differences in the number of vaginal spines between the two collections. The material collected from the Arabian Sea have 20–25 spines (25 spines on paratype ZIRAS acc. no. 12296) each measuring 48–54 in length, whilst the specimens collected for the current study have 16–18 spines, measuring 24–28 in length.

Each *Omanicotyle* specimen (18 individuals from 30 fish) was positioned on the outer hemibranch of the gill arch to which they were attached, orientated along the length of a single primary filament, the anterior end of the worm pointing towards the distal tip, with their clamps attached to the second lamellae; the posterior part of the haptor curling round the filament. The function of the numerous C2R positive droplets along the lateral margins of the anterior haptor is unknown and can only be speculated upon. Given their high number and aggregation, it is unlikely that these represent the precursors for the synthesis of new clamps, *i.e.* they are not in close association with developing clamps. It could be suggested that these function as papillae increasing the surface area of this part of the haptor and make a contribution to the attachment of the worm. Unfortunately, this area was not visible on the specimens prepared for SEM; further specimens are therefore required to determine whether these structures are visible externally. According to Mamaev [18], parasites belonging to the subfamily Microcotylinae Monticelli, 1892 have either a single medio-lateral or rarely a dorso-laterally positioned vagina or occasionally two dorso-laterally situated vaginae. Given the large size of the vaginae, which occupy almost the entire width of the worm, it cannot be said that they are strictly dorso-lateral or medial.

#### Differential diagnosis

*Omanicotyle* n. gen. can be differentiated from the other 24 genera in the subfamily Microcotylinae on the basis of vaginal number (single or paired) and its relative armature, and also by armature of the genital atrium. The species belonging to the following genera all possess a single, unarmed vagina: *Atriostella* Unnithan, 1971; *Caballeraxine* Lebedev, 1972; *Diplostamenides* Unnithan, 1971; *Gamacallum* Unnithan, 1971; *Jaliscia* Mamaev *et* Egorova, 1977; *Magniexcipula* Bravo-Hollis, 1981; *Paramicrocotyloides* Rohde, 1978; *Paranaella* Kohn, Baptista-Farias *et* Cohen, 2000; *Pauciconfibula* Dillon *et* Hargis, 1965 (syn. *Bradyhaptorus* Unnithan, 1971); *Polymicrocotyle* Lamothe-Argumedo, 1967; *Pseudoaspinatrium* Mamaev, 1986; *Sciaenacotyle* Mamaev, 1989 (single opening with paired vaginal ducts); and, *Solostamenides* Unnithan, 1971. Whilst those belonging to *Monomacracanthus* Mamaev, 1976 and *Sebasticotyle* Mamaev *et* Egorova, 1977 possess a single, armed vagina. Species belonging to the genus *Microcotyle* van Beneden *et* Hesse, 1863, also have a single, typically mid-dorsally positioned, vagina that is unarmed although the genus also includes species with an armed vagina (*e.g. Microcotyle pamae* Tripathi, 1954; see [19]). The remaining genera in the subfamily possess paired vaginae. Species belonging to the following genera have unarmed vaginae: *Bivagina* Yamaguti, 1963 (certain species within the genus); *Diplasiocotyle* Sandars, 1944; *Lutianicola* Lebedev, 1970; *Neobivagina* Dillon *et* Hargis, 1965 [based on *Neobivagina canthari* (van Beneden *et* Hesse, 1863) Dillon *et* Hargis, 1965 which was nominated by Mamaev [18] as the type species, following the reallocation of the other species in the genus to other genera]; *Pseudobivagina* Mamaev, 1986; and *Pseudoneobivagina* Mamaev, 1986. The remaining genera, which also include *Omanicotyle* n. gen., all have armed vaginae, *i.e. Bivagina* Yamaguti, 1963 (certain species within the genus); *Kahawaia* Lebedev, 1969; and *Neobivaginopsis* Villalba, 1987. *Omanicotyle* n. gen. can be readily discriminated from the other genera. The vaginae in *Bivagina* are typically small but heavily muscularised structures armed with a crescent of short spines; the vaginal ducts connecting the two vaginae are typically clearly visible. *Kahawaia* possesses two cuticularised, pyriform pads armed with spines, interpreted as vaginae. The vaginae of *Neobivaginopsis* are large, muscular, contractile structures the openings of which have lightly sclerotised borders. The vaginae of *Omanicotyle* n. gen. by comparison, are large, muscular structures with a full corona of spines, which near abut against each other and occupy almost the entire width of the worm and as such obscure the vaginal ducts (which could not be seen in the specimens examined for the current study).

*Omanicotyle* n. gen. can also be discriminated from almost all the other genera on the relative armature of the genital atrium and/or that of the cirrus/penis when present. The following genera have both an armed genital atrium and an armed cirrus: *Caballeraxine*; *Diplostamenides*; *Lutianicola*; *Neobivagina*; *Neobivaginopsis*; *Pseudobivagina*; *Pseudoneobivagina*; *Sciaenacotyle*; and *Sebasticotyle*. *Atriostella* possesses an armed genital atrium but an unarmed cirrus, whilst species belonging to the genera *Diplasiocotyle*, *Kahawaia*, *Jaliscia*, *Microcotyle*, *Paranaella*, *Polymicrocotyle* and *Solostamanides* possess an armed genital atrium but no differentiated cirrus. The genera *Gamacallum*, *Magniexcipula* and *Monomacracanthus* have an unarmed genital atrium but an armed penis/cirrus, whilst species belonging to *Paramicrocotyloides*, *Pauciconfibula* and *Pseudoaspinatrium* have both an unarmed genital atrium and an unarmed cirrus. Only the genera *Bivagina* and *Omanicotyle* n. gen. have an unarmed genital atrium and no differentiated cirrus. The latter two genera, however, can be readily separated on the size and armature of their vaginae, as discussed above.

#### Molecular results

**Figure 5:**
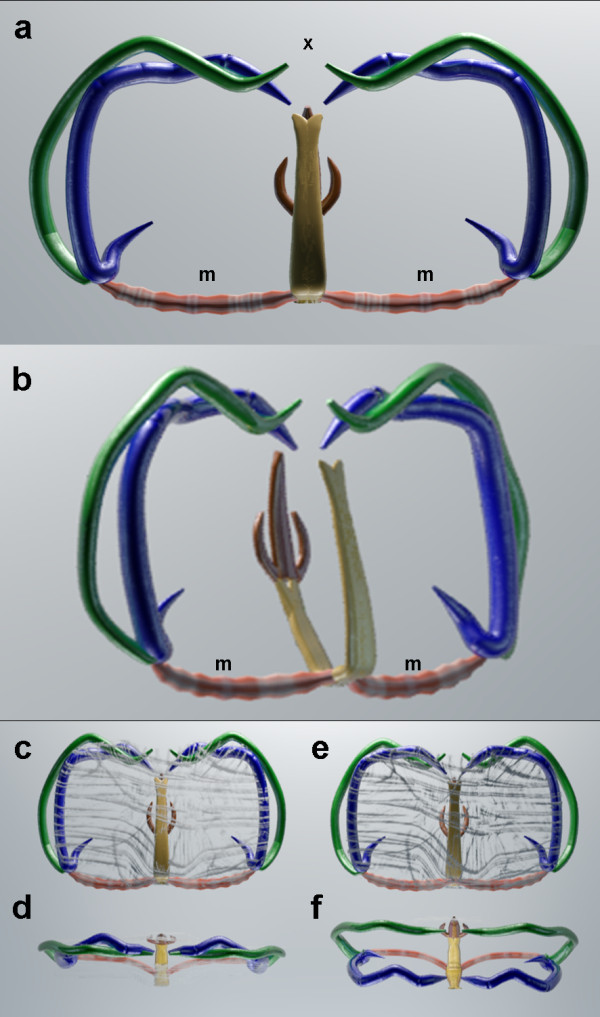
**Graphics representing the main components of the attachment clamps of *Omanicotyle heterospina *n. gen. *et *n. comb. a**, Median view. The clamp is in an upright position, the sclerites on the edge marked “x” open and attach to the secondary lamellae of the host’s gills. Large muscles (m) linking the sclerites, whilst others (not shown) run throughout the tegument that encases the entire clamp apparatus. **b**, medio-lateral view. **c**, median view of a clamp in the closed or “clamped” position. Supporting muscles that run through the tegument are shown. **d**, medio-ventral view of a closed clamp; **e**, median view of a clamp in the open position. **f**, medio-ventral view of an open clamp.

Both the SSU and LSU regions of the rDNA were successfully sequenced. A nucleotide BLAST search showed that the SSU rDNA of *O. heterospina* n. gen. *et* n. comb. was most similar to *Microcotyle sebastis* with a 99% identity, and the LSU rDNA was most similar to *B. pagrosomi*, both polyopisthocotylean monogeneans belong to the family Microcotylidae. The phylogenetic analyses produced similar tree topologies (Figure 5), *O. heterospina* n. gen. *et* n. comb. consistently grouped with *B. pagrosomi* as a strongly supported sister group to the *Microcotyle* clade.

## Discussion

There are 24 genera in the subfamily Microcotylinae [17], which, according to Mamaev [18] includes microcotylids that possess a symmetric or sub-symmetric, well-delineated haptor, adults that lack haptoral anchors, intestinal limbs with lateral branches/appendages without anastomoses, an armed or unarmed genital atrium, and, usually a single, medio-lateral, vagina, rarely latero-dorsal, but occasionally two, dorso-laterally or ventro-laterally positioned, vaginae. The subfamily Microcotylinae was revised by Unnithan [20] to include six new genera (*Atriostella*, *Diplostamenides*, *Manterella* Unnithan, 1971, *Nudimasculus* Unnithan, 1971, *Polynemicola* Unnithan, 1971 and *Solastamenides* Unnithan, 1971), alongside the four existing genera *Metamicrocotyla* Yamaguti, 1953, *Microcotyle* van Beneden *et* Hesse, 1863, *Microcotyloides* Fujii, 1944 and *Prosomicrocotyla* Yamaguti, 1958. This was subsequently revised by Mamaev [18] who moved *Polynemicola* and *Microcotyloides* to the subfamily Polynemicolinae Mamaev, 1986; *Metamicrocotyla* to the subfamily Metamicrocotylinae Yamaguti, 1963; *Prosomicrocotyla* to the subfamily Prosomicrocotylinae Yamaguti, 1963. Despite their removal from the Microcotylinae, however, no additional comment on the placement of *Manterella* and *Nudimasculus* was made. Mamaev [18] then moved a further 14 genera (*Bivagina*, *Caballeraxine*, *Diplasiocotyle*, *Gamacallum*, *Jaliscia*, *Kahawaia*, *Lutianicola*, *Magniexcipula*, *Monomacracanthus*, *Neobivagina*, *Neobivaginopsis*, *Paramicrocotyloides*, *Pauciconfibula*, *Polymicrocotyle*, *Pseudoaspinatrium*, *Pseudobivagina*, *Pseudoneobivagina* and *Sebasticotyle*) into the Microcotylinae. The genera *Paranaella*[21] and *Sciaenacotyle*[22] were subsequently added.

**Figure 6:**
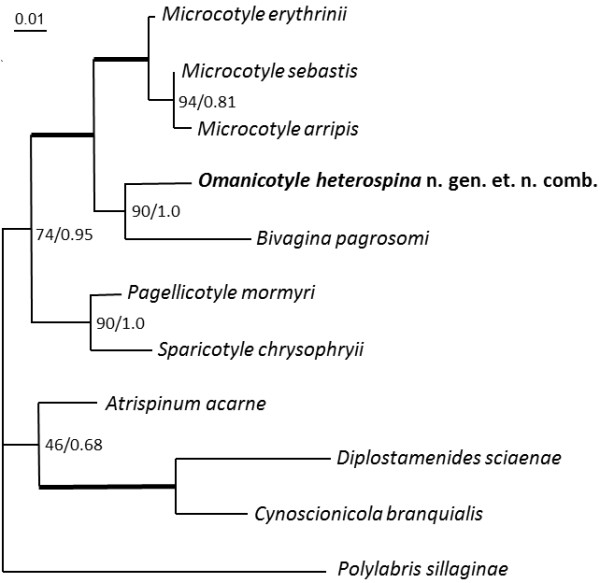
**Maximum likelihood phylogenetic tree for the Microcotylidae, based on 11 taxa and 947 characters of aligned LSU rDNA sequence data.** Non-parametric bootstrap values and Bayesian inference posterior probabilities are shown at the nodes. Bold branches lead to a node with a high bootstrap support of ≥95 and a Bayesian posterior probability of ≥0.98. The tree is rooted to the outgroup *Polylabris sillaginae* (Woolcock, 1936) Dillon, Hargis *et* Harrises, 1983. Scale bar represents 0.01 base changes per 100 bases.

The new genus *Omanicotyle* gen. n. bears microcotylid-like clamps each with a supplementary lanceolate process (see [18]; Figures 1d, 3c,d, and 5a-f), lack haptoral anchors in the adult, and have intestinal crura of unequal lengths, with short, anterior lateral branches (Figure 1a). The extent of intestinal branching, however, is unknown as much of its length is obscured by vitellaria. No branching was seen in the haptoral peduncle region. *Omanicotyle* gen n. is erected principally on the basis of the structure and armament of its vaginae, which are evident as two large muscular organs (av. 110 μm wide) occupying almost the entire width of each specimen (Figures 1a,e and 2c,d). Each distinct vagina is armed with a complete ring of 16–18 robust spines, confirmed by their positive reaction to the C2R-based sclerite stain [5] (Figure 3d). While two vaginae are a feature for several genera across the subfamily, the complexity and the degree of variation observed in the armament of these requires supporting molecular studies to unambiguously place species within a genus. Unfortunately, the Microcotylidae are not well represented in the databases with SSU rDNA sequences, however, more data are available for the D1-D2 regions of the LSU rDNA. In addition, the LSU rDNA is known to allow better phylogenetic resolution among monogenean family groups than the SSU counterpart [10]. From Figure 6, it can be seen that *Omanicotyle heterospina* n. gen. *et* n. comb. is well supported as a member of the subfamily Microcotylinae and as a sister taxon to the *Microcotyle*, grouping with *B. pagrosomi* (percentage similarity 95.2%; identities = 876/920 (95%); gaps = 10/920 (1%)). Other parts of the phylogeny, however, are not well resolved; *Diplostamenides sciaenae* (Microcotylinae), for example, groups with *Cynoscionicola “branquialis”* (Anchoromicrocotylinae Bravo-Hollis, 1981), which is weakly associated with *Atrispinum acarne* (Atriasterinae Maillard *et* Noisy, 1979). Additional gene sequence data for more taxa are, therefore, required to fully resolve the phylogenetic relationships amongst the Microcotylidae. Unfortunately, molecular data are also lacking for the purportedly closely related sister taxa of *Bivagina*, *i.e. Pseudobivagina* and *Neobivagina*. All three genera, however, can be separated on the degree of the genital atrium armature. Dillon & Hargis [23] separated *Bivagina*, with an unarmed genital atrium, from *Neobivagina*, with an armed genital atrium. Later, Mamaev [18] separated out *Pseudobivagina*, possessing a muscular copulatory organ armed dorsally with a semi-crown of rib-like spines, covered with longer ribs arranged in a dome configuration on the walls of the genital atrium. The genital atria of both *Bivagina* and *Omanicotyle heterospina* n. gen. *et* n. comb. are both unarmed. Although Dillon & Hargis [23] commented on the sclerotised nature of the cirrus (= penis sclerite in [4]) of another *Bivagina* species, *B. sillaginae* (Woolcock, 1936) Yamaguti, 1963 [syn. *M. sillaginae* Woolcock, 1936], they suggested this was atypical and as such the possible basis for its removal from the genus. This was subsequently moved and in Mamaev’s [18] revision of the Microcotylidae, this species as *Polylabris sillaginae* (Woolcock, 1936) has been placed within the subfamily Prostatomicrocotylinae Yamaguti, 1968. The complete armament of the vaginae, however, remains a key feature separating *Bivagina* species from *Omanicotyle heterospina* n. gen. *et* n. comb. Examination of *M.* [*Bivagina*] *centrodonti* and *B. pagrosomi* type material, photographs of *B. tai* provided by Professor K. Ogawa, and drawings provided in the literature [23-25] suggest that the proportionately small vaginae are predominantly armed with unequal sized spines in their lateral corners whilst the vaginae of *Omanicotyle heterospina* n. gen. *et* n. comb. are large, occupying the entire width of the worm and are armed with a full crown of robust, equal-sized spines. One additional feature that may be unique to *Omanicotyle* n. gen., which may have taxonomic significance, is the consistent, circular region of folded tegument on the dorsal surface (Figure 4a). Likewise, it is not known whether numerous C2R-positive (*i.e.* suggesting they are of the same proteins forming the hook material) regions distributed along the margins of the anterior portion of the haptor are unique and a definitive statement on these must wait until a detailed confocal microscopy-based study on representative species from each genus in the Microcotylinae can be conducted. The function of both these latter structures also requires confirmation but must await the collection of further specimens.

Although the number of monogeneans encountered in the current study was low, it is not known what impact these polyopisthocotyleans may pose to stock when *A. spinifer* is reared under intensive aquaculture conditions*.* Given the recorded pathogenicity of other microcotylids on captive held fish stocks (*e.g. Bivagina tai*[24,26]; *Microcotyle sebastis*[27]; *Sciaenacotyle panceri* (Sonsino, 1891) [28]; *Sparicotyle chrysophrii* (van Beneden *et* Hesse, 1863) Mamaev, 1984 [29]; and *Zeuxapta seriolae* (Meserve, 1938) [29-31]), a full assessment of the potential impact *Omanicotyle heterospina* n. gen. *et* n. comb. may have is advised before production is initiated.

## Conclusions

The polyopisthocotylean *Omanicotyle heterospina* n. gen. *et* n. comb. collected from the gills of the sparid *A. spinifer* is the first monogenean to be described from the Sea of Oman and is assigned to a new genus within the subfamily Microcotylinae (Microcotylidae), based on consistent morphological and molecular differences discriminating it from other genera in the subfamily. Morphologically, *Omanicotyle* n. gen., which possesses two large, fully-armed vaginae, can be discriminated from the genus *Bivagina* (*e.g. B. pagrosomi*) on differences in the armament of the vaginae and a characteristic circular, dorsal region of folded tegument. Sequencing of the LSU rDNA (949 bp) revealed only a 95.2% percentage similarity with *B. pagrosomi* (identities = 876/920 (95%); gaps = 10/920 (1%)), lending support to the proposal that these specimens are placed as a new taxon within the Microcotylinae. A full assessment of the disease potential of *Omanicotyle heterospina* n. gen. *et* n. comb. and how it may impact on the production of *A. spinifer* in Omani waters is advised before production begins.

## Competing interests

The authors declare that they have no competing interests.

## Authors’ contributions

GHY and SAJ sampled the fish, processed the monogeneans for light and scanning electron microscopy (SEM), took photographs and prepared specimens for evaluation by other methods. MAF conducted the molecular analyses. JEB examined specimens with the laser scanning confocal microscope and produced the graphics of the clamps. GP took morphometric measurements, made line drawings and co-defined, with APS, the differential diagnosis. APS prepared and examined specimens using confocal and SEM, made morphometric measurements and line drawings, identified the monogeneans, drafted the species description and the differential diagnosis. GHY, MAF, GP and APS drafted the manuscript. All authors read and approved the final version of the manuscript.
